# Cytotoxic Potential Evaluation of Innovative Pressurised Cyclic Solid–Liquid Extracts from *Withania somnifera*

**DOI:** 10.3390/plants15071027

**Published:** 2026-03-26

**Authors:** Rosanna Culurciello, Karen Power, Sergio Esposito, Ilaria Di Nardo, Simone Landi, Gionata De Vico, Domenico Palatucci, Elio Pizzo, Daniele Naviglio, Armando Zarrelli

**Affiliations:** 1 Department of Biology, University of Naples Federico II, Via Cintia 4, I-80126 Naples, Italy; rosanna.culurciello@unina.it (R.C.); karen.power@unina.it (K.P.); sergio.esposito@unina.it (S.E.); ilaria.dinardo@unina.it (I.D.N.); simone.landi@unina.it (S.L.); gionata.devico@unina.it (G.D.V.); domenico.palatucci@unina.it (D.P.); elipizzo@unina.it (E.P.); 2 Department of Chemical Sciences, University of Naples Federico II, Via Cintia 4, I-80126 Naples, Italy; daniele.naviglio@unina.it

**Keywords:** *Withania somnifera* (L.) Dunal, innovative pressurized cyclic solid–liquid extractor, withaferin A, withanolides, cytotoxic activity, tumour cell lines, cell viability inhibition, dose-dependent response

## Abstract

Ethnopharmacological relevance. *Withania somnifera* (L.) Dunal, widely used in traditional medical systems such as Ayurveda, Unani, and Middle Eastern folk medicine, is valued for its adaptogenic, anti-inflammatory, neuroprotective, antimicrobial, and anticancer properties. These activities are primarily attributed to withanolides, with Withaferin A recognized as one of the most bioactive constituents. Although traditional preparations often rely on the root, leaf use provides a more sustainable alternative and may yield significant quantities of active metabolites. Identifying efficient, modern extraction technologies that can enhance the recovery of bioactive compounds from leaves is essential for developing effective, standardized ethnopharmacological formulations. Materials and methods. Plants of *W. somnifera* grown from seeds were subjected to different environmental conditions (control, drought, cold, yeast extract treatment). Leaves were extracted using Pressurized Cyclic Solid–Liquid Extraction (PCSLE) with hydroalcoholic solvents and compared with conventional infusion of dried leaves. Extracts were fractionated with solvents of varying polarity and analyzed by TLC, HPLC, and NMR for quantification of Withaferin A. Expression levels of key withanolide-biosynthetic genes (CAS, SMT1, DWARF1, CYP71, CYP76) were assessed using qRT-PCR. Antimicrobial activity of pure Withaferin A, aqueous extract, and hydroalcoholic PCSLE extract was evaluated through MIC and MBC assays against Gram-positive and Gram-negative strains. Cytotoxic activity was measured via MTT assays in six human cancer cell lines after 3, 6, and 24 h of treatment. Results. PCSLE yielded substantially higher levels of Withaferin A than traditional infusion, especially in medium-polarity fractions (chloroform and ethyl acetate), with concentrations reaching 0.70% in fresh leaf mass (4.8% dry weight), compared to 0.11% obtained by infusion. Gene expression analysis revealed that 24-week-old plants exhibited the highest transcription of withanolide-biosynthetic genes, and drought stress significantly upregulated CAS, SMT1, DWARF1, CYP71, and CYP716, indicating enhanced metabolic flux toward withanolide production. Hydroalcoholic PCSLE extracts showed broad-spectrum antimicrobial activity, with MIC and MBC values comparable to pure Withaferin A and demonstrating bactericidal effects against *Pseudomonas aeruginosa*, *Escherichia coli*, *Staphylococcus aureus*, and *Listeria monocytogenes*. The aqueous extract showed activity only against Gram-positive strains. Cytotoxicity assays demonstrated an optimistic, dose-dependent reduction in cell viability across all tumour cell lines treated with the hydroalcoholic PCSLE extract, closely mirroring the activity of pure Withaferin A and consistently exceeding the effect of the aqueous extract. IC50 values confirmed the high bioactive content of PCSLE extracts and suggested mechanisms like those known for Withaferin A. Conclusions. PCSLE proved to be a highly efficient extraction technology for obtaining leaf extracts rich in Withaferin A, outperforming conventional extraction methods while exploiting sustainable plant tissue. Developmental stage and drought stress significantly modulated the expression of genes involved in withanolide biosynthesis, highlighting agronomic strategies capable of enhancing metabolite production. Hydroalcoholic PCSLE extracts exhibited antimicrobial and cytotoxic activities comparable to pure Withaferin A, supporting their relevance as promising therapeutic candidates. These findings advocate for the use of *W. somnifera* leaves as a sustainable source of bioactive compounds and demonstrate that advanced extraction technologies can contribute to the development of innovative ethnopharmacological preparations for antimicrobial and anticancer applications.

## 1. Introduction

**Figure 1:**
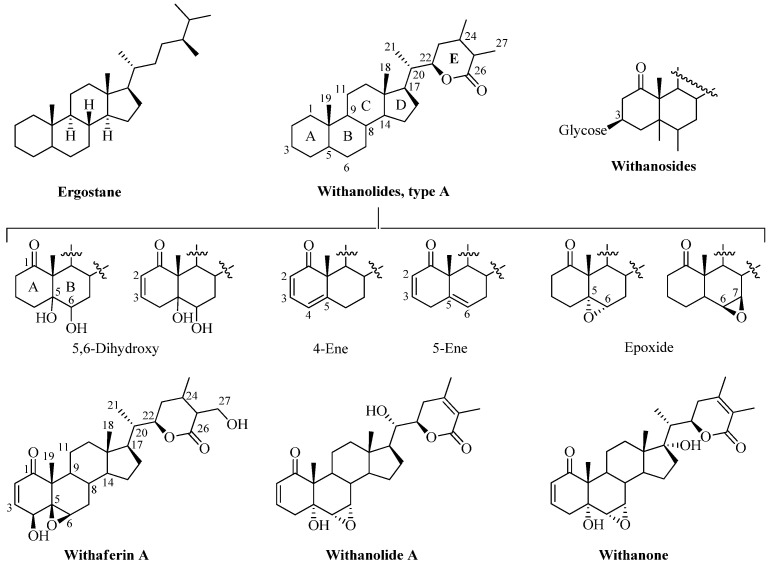
Chemical structures of withanolides, withanosides, and some of the best known withanolides.

*Withania somnifera* (L.) Dunal, 1852 (Ashwagandha) is a medicinal plant of the *Solanaceae* family [1] widely used in traditional Asian medical systems [2,3], especially Ayurveda [4,5]. Its extracts display anti-inflammatory, immunomodulatory, antitumoral, anxiolytic, and neuroprotective properties [5,6]. These activities are attributed to a diverse array of bioactive metabolites—over 80 identified—mainly produced in its roots—including alkaloids, steroids, flavonoids and phenolic compounds [5,6,7,8,9,10]. The most important class of molecules produced by *W. somnifera* consists of non-steroidal lactones (saponins) known as withanolides, of which more than 40 have been identified [5]. Withanolides share a C28-ergostane skeleton, in which carbons C26 and C22/C23 are oxidized to form lactone ring (Figure 1). Additional ring formation and/or modifications of the basic skeleton by chemical groups give rise to a wide array of molecular structures and biological activities [1,5,11,12]. Major withanolides include Withaferin A, Withanolide A, and Withanone. Among these bioactive compounds, Withaferin A has emerged as the most promising molecule, making it a potential candidate for the development of antitumoral drugs [13]. Notably, the specific biosynthetic pathway leading to withanolide formation has not yet been fully elucidated [5,14]. Several studies suggest that abiotic stress in *W. somnifera* may positively regulate the biosynthesis of secondary metabolites [1,15,16]. In particular, under water-limited conditions, *W. somnifera* exhibits significant physiological and metabolic adaptations that divert carbon flux toward withanolide the biosynthetic pathway(s) [1].

The aim of this study was therefore to evaluate the efficiency of Pressurized Cyclic Solid–Liquid Extraction (PCSLE) [17] in producing Withaferin A-rich extracts from *Withania somnifera* leaves, and to compare PCLSE extracts with those obtained by conventional infusion. In addition, we investigated how developmental stage and abiotic stress influence the expression of key withanolide-biosynthetic genes and assessed the in vitro antimicrobial and cytotoxic properties of PCSLE extracts relative to pure Withaferin A. These complementary approaches provide a comprehensive characterization of leaf-derived withanolides and support their potential relevance for further phytochemical and biotechnological studies.

## 2. Methods

### 2.1. Plant Materials and Treatments

Seeds of *W. somnifera* were supplied by the Botanical Gardens of the University “Federico II” of Naples and germinated for 10–14 days in the dark on moistened filter paper. Seedlings were then transferred to a greenhouse and grown in plastic pots (20 cm diameter) under controlled conditions (16–25 °C, 60–80% relative humidity). Leaves were collected at different developmental stages (from 6- to 48-week-old plants) and immediately frozen in liquid nitrogen for molecular analyses. Twenty-four-week-old *W. somnifera* plants were subjected to different stress treatments. Control plants (CTRL) were maintained at 18–25 °C and fully irrigated. For drought stress, plants were kept at 20–22 °C and irrigation was suspended for 2 weeks. For cold stress, plants were grown at 4 °C for 1 week with regular irrigation. Finally, for yeast elicitation, leaves were brushed with a 1% (*w*/*v*) yeast extract aqueous solution for three consecutive days.

### 2.2. Reagents and Materials

Withaferin A (98%) was purchased from ABCR GmbH (Karlsruhe, Germany). HPLC grade solvents and chem reagents were obtained from Sigma-Aldrich (Saint-Quentin Fallavier, France) and used without further purification. Deuterated solvents, including water, dimethyl sulphoxide, methanol, and chloroform were purchased from Sigma-Aldrich (Milan, Italy). Ultrapure water used in all experiments was produced using a Milli-Q purification system (Millipore, Milan, Italy).

### 2.3. Sample Preparation

**Scheme 1:**
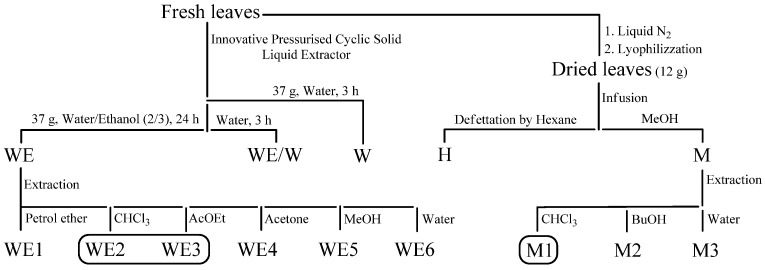
Fractionation scheme of the different extracts.

A total of 37 g of fresh leaves were extracted using an Innovative Pressurized Cyclic Solid Liquid Extractor (Atlas Filtri, Limena (PD), Italy) [17,18]. The extraction was first performed with a water:ethanol solution (2:3, *v*/*v*, 540 mL) and subsequently with water (540 mL), operating at a pressure of approximately 10 atm followed by rapid decompression to 0–1 atm (Scheme 1). An additional batch of 37 g of fresh leaves was extracted directly with water (540 mL). Each extraction cycle consisted of a 3 min static phase followed by a 1 min dynamic phase. The total extraction time was 24 h for the hydroalcoholic extract and 3 h for the aqueous extract. The hydroalcoholic extract (WE, 1.35 g) and the two aqueous extracts (WE/W and W, 0.36 and 0.61 g, respectively) were first partially concentrated using a rotavapor (Lyovapor L-200, Buchi, Cornaredo (MI), Italy) and then freeze-dried. The hydroalcoholic crude extract was subsequently fractionated with solvents of increasing polarity, as illustrated in Scheme 1, yielding fractions WE1–WE6.

In addition, 86 g of fresh leaves were frozen in liquid nitrogen, finely ground with a mortar, and freeze-dried, resulting in 12 g of dry leaf material. This material was first defatted with hexane (120 mL, 3 h) and then extracted with methanol (120 mL, 24 h). The methanolic extract (M, 2.9 g) was evaporated to dryness and subsequently fractionated with chloroform, butanol, and water to obtain fractions M1-M3 (0.59, 0.33 and 1.7 g, respectively).

TLC comparison with known and commercially available withanolides, together with HPLC and NMR analyses, revealed that the fractions containing withanolides were WE2, WE3 and M1. Fractions WE2 and WE3 were combined, and an aliquot of this pooled sample, as well as an aliquot of fraction M1, was accurately weighed, dissolved in CHCl_3_ in a 2 mL volumetric flask, and subjected to HPLC analysis for the determination of Withaferin A content.

### 2.4. Determination of Total Phenolic Content (TPC)

The total phenolic content of *W. somnifera* leaf extracts obtained with water, water/ethanol (2:3, *v*/*v*), and methanol was quantified using the Folin–Ciocalteu colorimetric method [19]. Briefly, an aliquot of each extract was mixed with the Folin–Ciocalteu reagent, and—after a reaction time of 5 min—the sodium carbonate solution was added to alkalinize the medium. The mixtures were then incubated in the dark at room temperature for 90 min to allow the development of the characteristic blue coloration. Absorbance was measured at 765 nm using a Thermo Scientific Varioskan Lux spectrophotometer.

Gallic acid was used as the calibration standard, and the results were expressed as mg gallic acid equivalents (GAE) per g of extract. All analyses were performed in triplicate.

### 2.5. Determination of Withaferin a Content by HPLC

**Figure 2:**
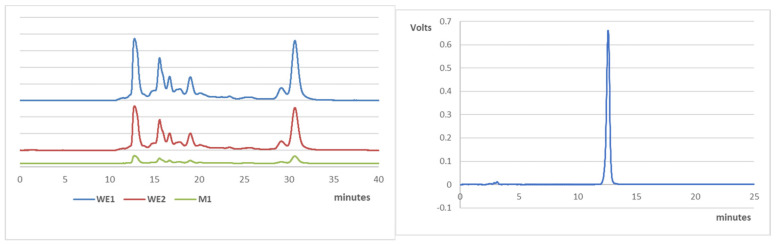
HPLC chromatograms of the plant extracts and of Withaferin A.

The determination of Withaferin A was carried out using a Shimadzu LC-9A HPLC system equipped with a Shimadzu SPD-6A UV–Vis detector (Shimadzu Italia S.r.l., Milan, Italy) and an RP18 analytical column (Gemini, 5 μm particle size, 150 mm × 4.6 mm i.d., Phenomenex, Bologna, Italy). Quantification was performed under isocratic conditions using a mobile phase consisting of acetonitrile and 0.1% formic acid in water (35:65, *v*/*v*) at a flow rate of 1.0 mL/min. UV detection was set at 230 nm. One millilitre of each extract was appropriately diluted with methanol, and an accurately measured aliquot was analyzed by HPLC, in triplicate. A calibration curve, constructed using six different concentrations of Withaferin A, was used for quantification. A representative chromatogram is shown in Figure 2.

### 2.6. RNA Extraction and qRT-PCR

RNA was extracted from 100 mg of fresh leaves using TRizol reagent (Life Technologies, Carlsbad, CA, USA). RNA quantity and purity were assessed using a NanoDrop ND-1000 spectrophotometer (Thermofisher, Waltham, MA, USA). cDNA synthesis was performed using the ThermoScript RT-PCR System. Gene expression analysis was conducted by qRT-PCR using Platinum SYBR Green qPCR SuperMix (Life Technologies, Carlsbad, CA, USA). CYCLOPHILLIN was used as an endogenous reference gene [20]. Relative gene expression levels were calculated using the 2^−ΔΔCt^ method as described by Livak & Schmittgen [21]. mRNA abundance in each sample was expressed relative to the calibrator, and results are reported as fold change (fc). The primer sequences used in this study are listed in Table 1.

### 2.7. Antimicrobial Assay (MIC, MBC)

The determination of the MIC values was performed on Gram(+) and Gram(−) bacteria using the broth microdilution method previously described for antimicrobial peptides in Cafaro et al. [22]. Assays were conducted in 0.5× Nutrient Broth (Difco, Detroit, MI, USA) using sterile 96-well polypropylene microtiter plates (Costar, cat. 3879; Cambridge, MA, USA). Bacterial strains were grown overnight in Luria–Bertani (LB) medium at 37 °C and subsequently diluted in Nutrient Broth to a final concentration of 1 × 10^6^ CFU/mL in each well. Twofold serial dilutions of Withaferin A, as well as of the aqueous and hydroalcoholic extracts of *W. somnifera*, were prepared directly in the wells to obtain final concentrations ranging from 12.5 mg/mL to 0.19 mg/mL. Ciprofloxacin and tobramycin were used as positive antimicrobial control [23], at the same experimental conditions. The microplates were then incubated overnight at 37 °C. The MIC value was defined as the lowest concentration that completely inhibited visible bacterial growth. Each MIC determination was performed in triplicate in three independent experiments.

MIC values were assessed against the Gram(−) strains *Pseudomonas aeruginosa* PAO1 and *Escherichia coli* ATCC 25922, and the Gram(+) strains *Staphylococcus aureus* ATCC 6538P and *Listeria monocytogenes* ATCC 7644. To evaluate the bactericidal activity of Withaferin A and of the aqueous and hydroalcoholic extracts, the MBC was determined from the MIC broth dilution series by subculturing aliquots onto LB agar plates. The MBC was defined as the lowest concentration capable of killing ≥ 99.9% of the initial bacterial population.

### 2.8. Cell Culture and Treatments

SH-SY5Y, U87MG, MCF-7, HepG2, Caco2, and HeLa cells were cultured in Dulbecco’s Modified Eagle Medium (DMEM) supplemented with 10% fetal bovine serum (FBS), 1 mM L-glutamine, and 1% (*v*/*v*) penicillin/streptomycin solution (100 U/mL). Cells were maintained at 37 °C in a humidified atmosphere containing 5% CO_2_.

Cells were treated with increasing concentrations of Withaferin A, aqueous extract and hydroalcoholic extract of *W. somnifera*, up to 2 mg/mL, for different incubation times depending on the assay.

### 2.9. Cell Viability Assay

For the cytotoxicity assay, 5 × 10^3^ cells were seeded in 96-well plate and, after 24 h of growth, were treated with increasing concentrations of Withaferin A, aqueous extract, or hydroalcoholic extract of *W. somnifera*. Cells viability was assessed after 3, 6 and 24 h of treatment using the MTT assay [24], and absorbance was measured at 570 nm with a Multi-Mode Microplate Reader (Santa Clara, CA, USA, Synergy™ H4).

### 2.10. Statistical Analysis

Statistical analyses were performed using GraphPad Prism 9.0.0 (Adalta, Arezzo, Italy). Data are reported as mean ± SEM from biological replicates in at least three independent experiments. Statistical significance was evaluated by one-way ANOVA followed by Bonferroni’s post hoc test. Significance levels were indicated as *p* < 0.05, *p* < 0.01, *p* < 0.001, and *p* < 0.0001 compared to the respective controls.

The half-maximal inhibitory concentration (IC_50_) values were determined by nonlinear regression analysis using a four-parameter logistic (4PL) dose–response model in GraphPad Prism 9.0.0. Data were fitted to a log(inhibitor) vs. normalized response model with variable slope. IC_50_ values were calculated automatically by the software from the fitted curves.

## 3. Results

### 3.1. Total Phenolic Content (TPC)

The Folin–Ciocalteu assay revealed marked differences in total phenolic content among the *W. somnifera* leaf extracts. The methanolic extract showed the highest value (90 ± 6.4 mg GAE g^−1^), followed by the aqueous extract (70 ± 2.1 mg GAE g^−1^), while the water/ethanol (2:3) extract displayed a substantially lower content (27 ± 4.3 mg GAE g^−1^). These differences reflect the solvent-dependent solubility of phenolic compounds, with polar organic solvents such as methanol extracting a broader range of polyphenols. Although phenolics are not directly involved in withanolide biosynthesis, their quantification provides useful complementary information, as they may contribute to the overall antioxidant and biological activity of the extracts. The lower TPC in the hydroalcoholic PCSLE extract suggests that this technique preferentially enriches non-phenolic constituents, such as withanolides, while still allowing a partial recovery of phenolic compounds.

### 3.2. Determination of Withaferin a Content

Withaferin A content was determined by HPLC in fresh leaves extracted using an Innovative Pressurized Cyclic Solid Liquid Extractor, and in dried leaves extracted by infusion. The extracts containing the highest amounts of Withaferin A were those of medium-low polarity: the chloroform (WE2) and ethyl acetate (WE3) fractions obtained from the hydroalcoholic PCSLE extract (WE), and the chloroform fraction (M1) derived from the methanolic infusion extract (M).

Specifically, fractions WE2-WE3 contained an estimated 0.70% Withaferin A relative to fresh leaf weight, corresponding to 4.8% of dry leaf weight. In contrast, the M1 fraction obtained from the infusion contained approximately 0.11% Withaferin A relative to fresh leaf weight, corresponding to a 0.79% of dry leaf weight.

### 3.3. Expression Analysis of Genes Involved in Withanolide Pathway

**Figure 3:**
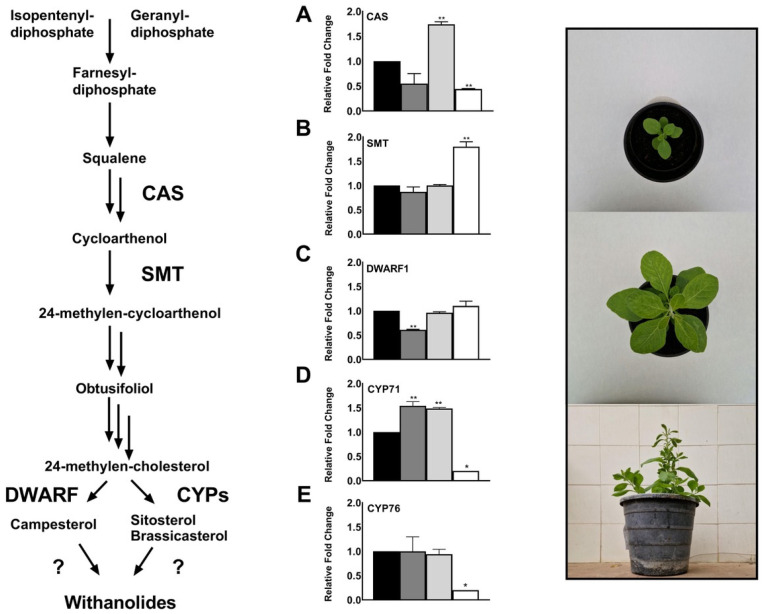
Changes in the gene expression (qRT-PCR) of CAS (**A**), SMT(**B**) DWARF (**C**), CYP71 (**D**) and CYP76 (**E**) in *Withania* leaves from plants grown in CTRL condition after 6 weeks (black bars), 8 weeks (dark grey bars), 24 weeks (light grey bars) and 48 weeks (white bars). mRNA levels were calculated relative to the expression of the Cyclophillin used as calibrator. Values were shown as fold change (-fc; ratio of the normalized expression value 8/24/48 weeks vs. 6 weeks). Asterisks showed a significance between stressed and the control plants. * = *p* < 0.05, ** = *p* < 0.005.

The expression of genes involved in withanolide biosynthesis (CAS, SMT1, DWARF1/Sterol C-24 Reductase, CYP71, and CYP76) was analyzed in leaves from plants at different developmental stages using qRT-PCR. Overall, the results indicated a general increase in transcript abundance in mature plants. As shown in Figure 3, CAS expression increased significantly in 24-week-old plants (+1.74-fold), whereas no significant changes were observed in 8 weeks. SMT1 expression showed a two-fold increase in 48-week-old plants. DWARF1 expression remained relatively constant up to 48 weeks, except for a significant decrease to 8 weeks. CYP71 expression increased significantly (~1.5-fold) in both 8- and 24-week-old plants, followed by a marked reduction at 48 weeks. Finally, CYP76 expression remained stable during the early developmental stages.

**Figure 4:**
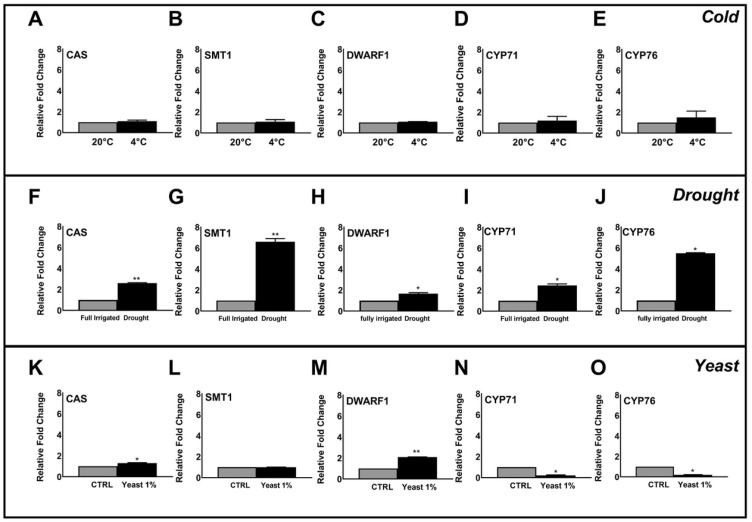
Changes in the gene expression (qRT-PCR) of CAS, SMT, DWARF, CYP71, and CYP76 in *Withania* leaves from plants grown in CTRL (black bars) and upon Cold (**A**–**E**), Drought (**F**–**J**) and Yeast (**K**–**O**) conditions (grey bars). mRNA levels were calculated relative to the expression of the Cyclophillin used as calibrator. Values were shown as fold change (-fc; ratio of the normalized expression value-treated plants vs. CTRL). Asterisks showed a significance between stressed and the control plants. * = *p* < 0.05, ** = *p* < 0.005.

Based on these observations, the 24-week developmental stage was selected for further analyses. Plants were subjected to different treatments (CTRL, Drought, Cold, and Yeast; Figure 4). Low-temperature stress produced only minor effects, with no significant changes detected in the expression of any analyzed genes (Figure 4A–E). In contrast, drought stress induced a clear and significant up-regulation of all genes examined: CAS (+2-fold), SMT1 (+7.37-fold), DWARF1 (+1.6-fold), CYP71 (+2.4-fold), and CYP716 (+5.5-fold) (Figure 4F–J). Yeast elicitation triggered a slight up-regulation of CAS (+1.3-fold) and DWARF1 (+2.1-fold). Interestingly, YEAST treatment caused a strong down-regulation of CYP71 (−4.7-fold) and CYP76 (−4.1 fold) (Figure 4K–O).

Based on these results, CTRL and drought-treated plants were selected for withanolide extraction and subsequent analyses.

### 3.4. Antimicrobial Activity

Withaferin A is commercially available, and the aqueous and hydroalcoholic extracts of *W. somnifera* were prepared as described in Section 2.3.

The antimicrobial activity of Withaferin A, the aqueous extract, and the hydroalcoholic extract of *W. somnifera* was assessed by determining their minimal inhibitory concentration (MIC) values against a panel of bacterial strains, including two Gram(−) species (*P. aeruginosa* PAO1 and *E. coli* ATCC 25922) and two Gram(+) species (*S. aureus* ATCC 6538P and *L. monocytogenes* ATCC 7644), including two antibiotics (ciprofloxacin and tobramycin) as control [23]. As reported in Table 2 the hydroalcoholic extract of *W. somnifera* showed antimicrobial activity against all strains tested, with MIC values generally around 3.125 mg/mL. In contrast, the aqueous extract showed limited activity, inhibiting exclusively Gram-positive strains, while no significant effect was observed against Gram-negative bacteria.

Minimum Bactericidal Concentration (MBC) values (Table 2) were identical or very close to the corresponding MIC values for Withaferin A and the hydroalcoholic extract, resulting in MBC/MIC ratios consistent with a bactericidal effect. In contrast, the aqueous extract showed significantly weaker bactericidal activity, with higher MBC values and less consistent bactericidal profiles.

The relatively lower antimicrobial activity of the aqueous extract may be related to differences in phytochemical composition associated with the polarity of the solvent. Due to its high polarity, water is less efficient at extracting lipophilic bioactive constituents, such as withanolides, which hypothetically contribute significantly to the antimicrobial properties of *W. somnifera*. Therefore, the reduced activity observed in the aqueous extract likely reflects a lower concentration of these compounds rather than an inherently weaker antimicrobial potential.

### 3.5. Cytotoxicity and Potential Anti-Cancer Activity of Withaferin A, Aqueous and Hydroalcoholic Extract of W. somnifera

**Figure 5:**
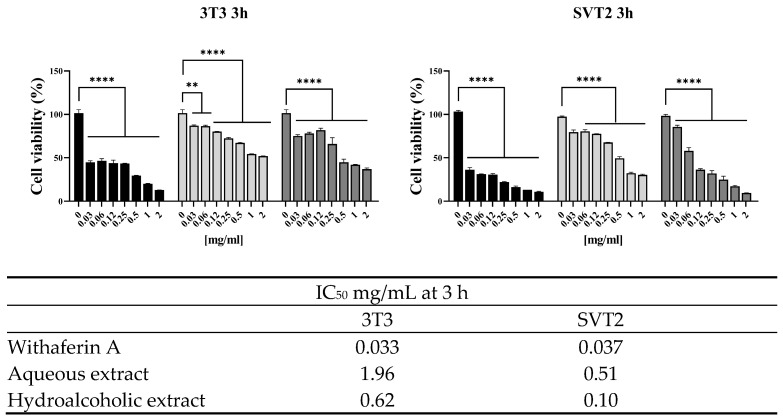
Cell viability analysis and relative IC_50_ on murine fibroblasts non-cancerous 3T3 and SVT2 tumour cells of Withaferin A (black), aqueous extract (light grey) and hydroalcoholic extract (dark grey) for 3 h. The experiments were performed in triplicate each on *n* = 3 biological replicates and reported as mean ± SEM. Statistical analyses were carried out by one-way ANOVA, followed by Bonferroni’s post-test (** *p* < 0.01 or **** *p* < 0.0001). IC_50_ for each sample was calculated by using nonlinear regression method. See Section 2 for experimental details.

The hydroalcoholic PCSLE, based on the “Naviglio principle” [17], allows the recovery of an extract enriched in bioactive compounds—in this case, Withaferin A. This observation supports the hypothesis that the hydroalcoholic PCSLE extract may possess enhanced or additional biological activities. To test this assumption, we compared the cytotoxicity of the PCSLE hydroalcoholic extract with that of the aqueous extract, using pure Withaferin A as a reference control. The cytotoxic effects of extracts were evaluated in murine fibroblast models, including non-cancerous 3T3 cells and SVT2 transformed fibroblasts (Figure 5). SVT2 cells showed greater sensitivity to the treatment, with viability reduced by up to 20% at the highest concentration tested, compared to 3T3 cells, where viability was not reduced by more than 50% under the same conditions. These results suggest a preferential effect on transformed cells and prompted further investigation in human cancer cell models.

**Figure 6:**
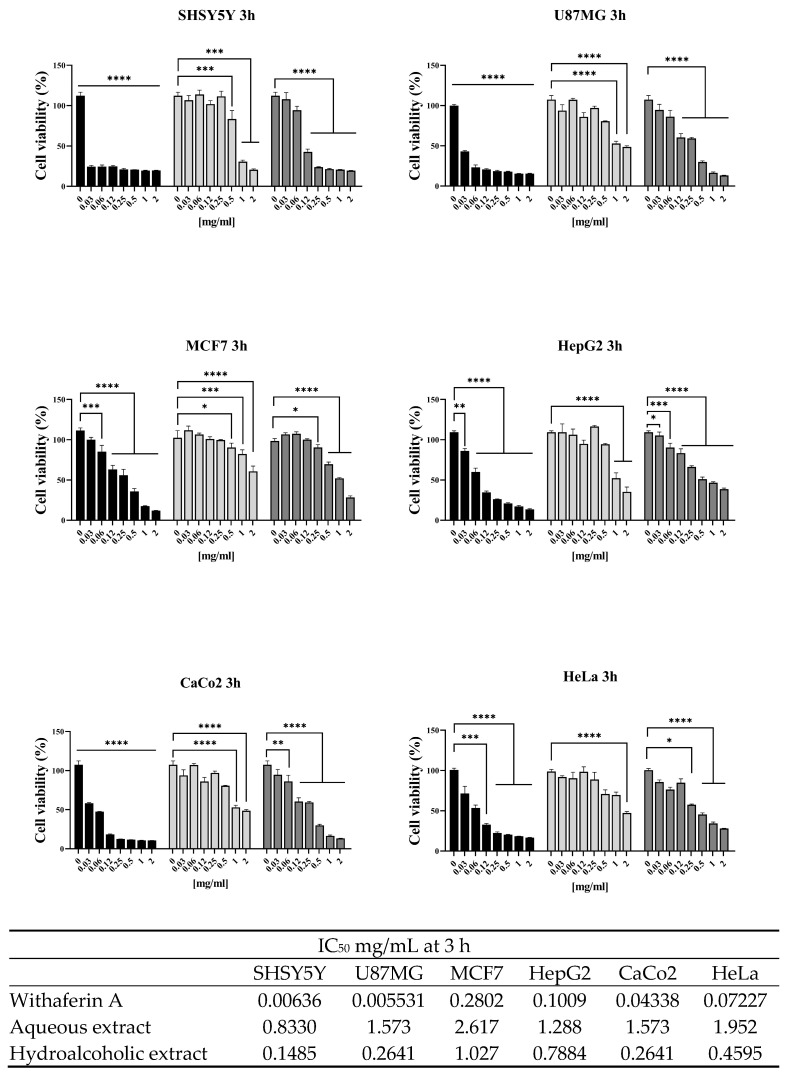
Cell viability analysis and relative IC_50_ on human cancer cells treated with serial dilutions of Withaferin A (black), aqueous extract (light grey) and hydroalcoholic extract (dark grey) for 3 h. The experiments were performed in triplicate each on *n* = 3 biological replicates and reported as mean ± SEM. Statistical analyses were carried out by one-way ANOVA, followed by Bonferroni’s post-test (* *p* < 0.05, ** *p* < 0.01, *** *p* < 0.001 or **** *p* < 0.0001). IC_50_ for each sample was calculated by using nonlinear regression method. See Section 2 for experimental details.

**Figure 7:**
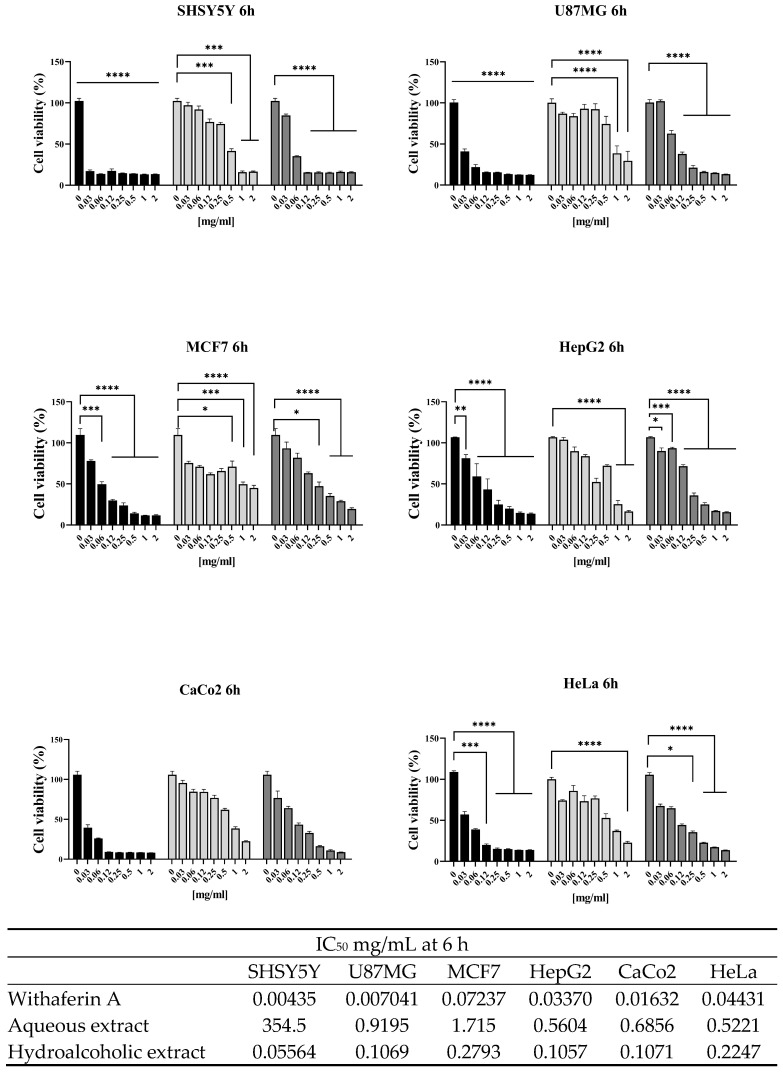
Cell viability analysis and relative IC_50_ on human cancer cells treated with serial dilutions of Withaferin A (black), aqueous extract (light grey) and hydroalcoholic extract (dark grey) for 6 h. The experiments were performed in triplicate each on *n* = 3 biological replicates and reported as mean ± SEM. Statistical analyses were carried out by one-way ANOVA, followed by Bonferroni’s post-test (* *p* < 0.05, ** *p* < 0.01, *** *p* < 0.001 or **** *p* < 0.0001). IC_50_ for each sample was calculated by using nonlinear regression method. See Section 2 for experimental details.

**Figure 8:**
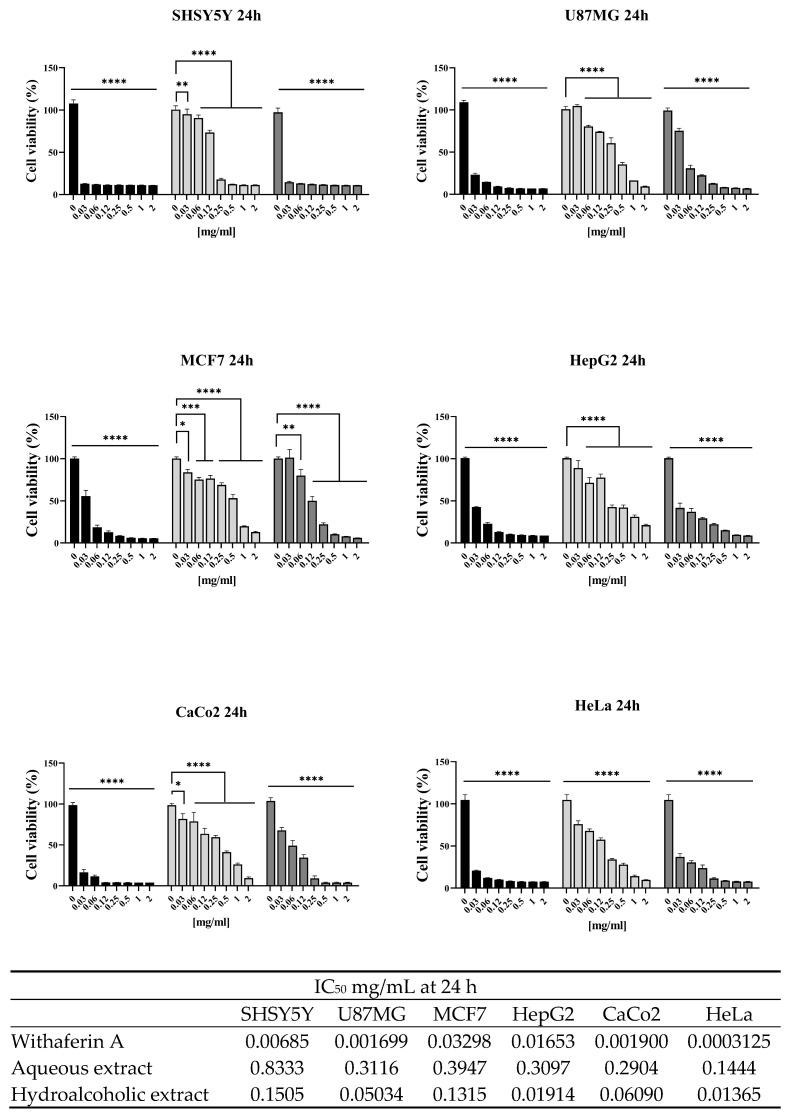
Cell viability analysis and relative IC_50_ on human cancer cells treated with serial dilutions of Withaferin A (black), aqueous extract (light grey) and hydroalcoholic extract (dark grey) for 24 h. The experiments were performed in triplicate each on *n* = 3 biological replicates and reported as mean ± SEM. Statistical analyses were carried out by one-way ANOVA, followed by Bonferroni’s post-test (* *p* < 0.05, ** *p* < 0.01, *** *p* < 0.001 or **** *p* < 0.0001). IC_50_ for each sample was calculated by using nonlinear regression method. See Section 2 for experimental details.

To this end, cell viability assays were performed on a broad panel of human tumour cells lines, using serial dilutions of the extracts. Analyses were conducted at different incubation times (3, 6 and 24 h), as shown in Figure 6, Figure 7 and Figure 8. The results (Figure 6), clearly demonstrate that the PCSLE hydroalcoholic extract exerts a marked, dose-dependent cytotoxic effect on all tested cell lines, with IC_50_ values closely resembling those observed for pure Withaferin A. Moreover, under experimental conditions (scaled doses and multiple incubation times; Figure 7 and Figure 8), the hydroalcoholic PCSLE extract consistently exhibited greater cytotoxicity than the aqueous extract. These findings reinforce the initial hypothesis that the PCSLE extract contains higher concentrations of active compounds and may display additional or enhanced bioactivities.

## 4. Discussion

The characterization of well-known plant secondary metabolites used in traditional medicine represents a valuable strategy for modern biotechnological research. In this study, *W. somnifera* plants were cultivated under different conditions and analyzed at multiple developmental stages to identify favourable parameters that enhance withanolide production for potential applications in disease-related cellular models. Based on the available literature, the PCSLE technique has not yet been reported for the extraction of bioactive compounds from *W. somnifera* leaves. Our study addresses this unexplored aspect by applying PCSLE to leaf material for the first time. It is worth noting that most of the major findings concerning the medical properties of *W. somnifera* have been obtained from root-derived bioactive compounds [2,3]. Root extracts have shown significant neuroprotective effects in mice by modulating apoptotic markers such as BCL2 and BAX [2], increasing dopamine levels in Parkinson’s disease models [3], and activating antioxidant enzymes [25]. The use of leaf extracts, however, offers a considerable advantage, as it avoids the destruction of entire *W. somnifera* plants and allows for sustainable harvesting strategies. Moreover, although several studies have described the pharmacological effects of *W. somnifera* extracts, surprisingly little is known about the specific biosynthetic pathways responsible for withanolide production in planta. Current evidence suggests that the early steps of the pathway overlap with sterol biosynthesis, while subsequent reactions involve specific cytochrome P450 enzymes [1,14].

This limited understanding underscores the need for deeper molecular and biochemical investigations to elucidate the regulatory mechanisms controlling withanolide accumulation. Our data showed an increased expression of sterol-related genes (CAS, SMT, DWARF1) in adult plants (24 weeks), suggesting an enhanced availability of an availability of withanolide precursors. Consistently, different chemoprofile studies have identified campesterol, brassicasterol, and stigmasterol as key intermediates in withanolide biosynthesis (4). Nevertheless, the critical steps underlying the conversion of sterols into specific withanolides (e.g., Withaferin A) remain largely unresolved. A sterol-C24-methyltransferase type 1 (SMT1) has recently been identified as a regulatory enzyme within this biosynthetic route [26], yet its precise role in the pathway requires further investigation. Interestingly, the expression of cytochrome P450 genes (CYP71 and CYP76) was drastically reduced in 4-week-old plants, suggesting a distinct regulatory pattern during late developmental stages. Such regulation may significantly influence the profile of withanolides produced. Indeed, plants overexpressing CYP71 exhibit reduced accumulation in Withanolide A, whereas overexpressing CYP76 enhances Withaferin-A biosynthesis and decreases levels of 12-deoxywithastramonolide and Withanolide A [14].

Together, these observations point to a complex developmental regulation of the pathway, with specific P450 enzymes playing central roles in defining the withanolide chemotype. Furthermore, we evaluated the effects of different stress conditions—drought, cold, and yeast elicitation—on withanolide biosynthesis. The activation of withanolide biosynthetic pathways under stress has previously been reported by several authors [1,15,16]. In agreement with these studies, our results showed that drought was the only condition capable of inducing a coordinated up-regulation of CAS, SMT, DWARF1, CYP71 and CYP76. This finding is consistent with transcriptomic and metabolomics datasets showing that drought stress enhances both withanolide accumulation (e.g., Withanoside V, Withastamonoside) and the expression of genes involved in sterol biosynthesis [1]. Yeast treatment resulted in an up-regulation of CAS and DWARF1, accompanied by a down-regulation of CYP71 and CP76. This pattern aligns with observations by Razdan et al. [27], who reported similar DWARF1 regulation in response to plant hormones and elicitors, highlighting the role of withanolides in mediating responses to biotic cues. Conversely, the suppression of P450-related genes suggests that different withanolides may participate in distinct physiological processes, each governed by specific regulatory mechanisms. Interestingly, leaf extracts revealed the exclusive presence of Withaferin A, a finding that underscores the unique chemotypic features of the *W. somnifera* genotype utilized in this study. Although the determination of Withaferin A content revealed minor differences between CTRL and stressed conditions (especially drought), the expression analyses on putative biosynthetic genes remain promising. The induction of genes such as CAS, SMT, DWARF1, CYP71, CYP76 and others could represent an effective strategy to increase the content of Withaferin and improve biological activity. Further studies should be necessary to define severe stress conditions that improve the quality of the leaf extracts but are not lethal for the plant.

These suggestions were recently supported by genetic engineering of related species, namely *Withania coagulans* [28]. Modification in the expression of the specific *rolA* gene of this species obtained by *Agrobacterium tumefaciens* transformation enhances secondary metabolite production, antioxidant activity, and anticancer activity of transformed tissues.

It is also possible that the biological activity of the PCSLE hydroalcoholic extract does not depend solely on its Withaferin A content. Several studies indicate that withanolides can act synergistically, enhancing cytotoxic or antimicrobial effects when present together rather than individually. Although Withanone was not detected in our extracts, other minor withanolides, sterols, or phenolic compounds may contribute to the overall activity profile. Indeed, despite phenolic content of leaf extract data being heterogeneous—they depend on extraction methods and on plant cultivation strategy [29,30], they support the hypothesis that synergistic effects may arise from the combination of different metabolite pharmacological activity. Such interactions may help explain why the PCSLE extract, while less active than pure Withaferin A in terms of IC_50_ values, still shows promising biological effects. Further fractionation studies will be needed to clarify these synergistic contributions.

The potential anticancer activity of *W. somnifera* compounds was then investigated across different human cancer cell lines to assess their potential antitumor properties. SH-SY5Y is a subclone of a neuroblastoma cell line widely used not only in cancer research but also in studies of neurodegenerative diseases. In this cell line, the results of our study are particularly relevant, as both pure Withaferin A and the PCLSE extracts displayed clear cytotoxic effects on the SH-SY5Y cell line. The hydroalcoholic leaf extract and Withaferin A induced cytotoxicity even at low concentrations and after only three hours of incubation. Previous studies by Widodo et al. [31,32] demonstrated that alcoholic extracts of *W. somnifera* leaves contain Withanone, one of the major active constituents of the plant, and they reported that the antineoplastic activity of leaf extracts is primarily attributable to Withanone. Conversely, in our extract Withanone was not detected, and the hydroalcoholic PCSLE extract suggests that the cytotoxic effect is mainly driven by Withaferin A, highly detected in the PCLSE hydroalcoholic extract.

The U87MG cell line is a human glioblastoma model widely used in cancer research, representing one of the most aggressive and therapeutically challenging brain tumours [33]. Both *W. somnifera* extracts and Withaferin A have been previously investigated in vitro in U87MG cells and multiple studies have demonstrated that Withaferin A exerts dose- and time-dependent cytotoxicity on this glioblastoma line [34]. Consistently with previous studies, in our experiments, Withaferin A exhibited a clear cytotoxic effect even at 0.03 mg/mL after only 3 h of exposure. *W. somnifera* extracts have likewise demonstrated antineoplastic activity in many in vitro and in vivo models [35]. In our study, both aqueous and hydroalcoholic leaf extracts showed cytotoxic activity, with the hydroalcoholic extract being particularly effective at 0.25 mg/mL after 24 h.

HeLa cells are widely used in cervical cancer research. *W. somnifera* extracts have also shown to exert dose- and time-dependent cytotoxicity on HeLa cells [36,37]. In line with these findings, both pure Withaferin A and hydroalcoholic PCSLE extract displayed marked in vitro toxicity toward HeLa cells in our experiments, without implying any direct therapeutic relevance.

Caco-2 cells, derived from human colorectal adenocarcinoma, are widely used in cancer, pharmacological, and toxicological research [38]. Although data regarding the effects of Withaferin A on Caco-2 cells remain limited, available studies indicate that Withaferin A can exert in vitro cytotoxic effects in colorectal cancer (CRC) cell lines [39].

While the cytotoxic effects of the Withaferin A in the examined cell lines appear clear, the potential mechanisms of action were not investigated. However, based on previous studies, we can speculate that the mechanisms through which Withaferin A exerts its antitumoural action include induction of apoptosis, inhibition of cell proliferation, and modulation of several signalling pathways involved in tumor progression [37,40].

Although additional studies are needed to elucidate the specific molecular pathways involved, these findings warrant further investigation which could open to potential therapeutic implications.

## 5. Conclusions

*Withania somnifera* was cultivated from seeds collected at the Botanical Garden of Naples. Fresh leaves were extracted using an Innovative Pressurized Cyclic Solid–Liquid Extractor (PCSLE), while dried leaves were extracted using conventional infusion methods with solvents of increasing polarity. The resulting extracts were analyzed for the presence of one of the most studied withanolides, specifically Withaferin A, which was quantified using HPLC. Extracts obtained with solvents of medium polarity (chloroform and ethyl acetate) contained the highest levels of Withaferin A, namely 0.70% by weight of the fresh leaves for the PCLSE extracts and 0.11% for the infusion extract. The data presented here underscore possible advantages of PCSLE over conventional extraction techniques in obtaining Withaferin A-rich extracts from *W. somnifera* leaves. The technique, particularly when combined with hydroalcoholic solvents, yield extracts with promising antimicrobial and cytotoxic activities in in vitro experiments. Although these extracts display interesting bioactivity, their potency remains lower than that of pure Withaferin A, as reflected in the IC_50_ values. Nevertheless, PCSLE extracts consistently outperformed aqueous extracts, highlighting the efficiency of the method. Developmental stage and abiotic stress—particularly drought—were found to influence the expression of key biosynthetic genes, supporting the potential of controlled cultivation practices to enhance Withanolide production. These insights highlight the value of integrating advanced extraction technologies such as PCSLE with targeted agronomic strategies. Moreover, the use of leaf material instead of roots could provide a sustainable and ecologically responsible approach to harnessing the medicinal potential of *W. somnifera*. Future research should focus on isolating additional bioactive compounds within PCSLE extracts and investigating their potential synergistic interactions with Withaferin A. Collectively, these findings offer a starting point in understanding the possible use of Withanolide compounds in new antimicrobial and antineoplastic formulations.

## Figures and Tables

**Table 1:** List of primers used for qRT-PCR.

Locus	Primer
WsCyp76Fw	AGCCTGAGAGGTTTCTTGGC
WsCyp76Rv	CAACACACATCCTCCGTCCA
WsCyp71Fw	GAGTTGCCGTGTGGAATGAA
WsCyp71Rv	TCTGGGAACAAGGCACAGAG
WsSMT1 Fw	GTGGGACTTGCTCCTAAAGGT
WsSMT1 Rv	TGGCGTGAAGATCCCTTTCT
WsDWF1 Fw	TTAAGGCCACCCAAAGCGA
WsDWF1 Rv	GAGGGTATCCGCAACCTTGT
WsCyclophillin Fw	AGGTCCAGCATTAGCCATCG
WsCyclophillin Rv	GAGAATCAATCTACGGCGCA
WsCAS Fw	GGTCCTATCACGCCAACAGT
WsCAS Rv	GCACACTCATTGCGAGCTTT

**Table 2:** Antimicrobial activity analysis. Evaluation of MIC (Minimum Inhibitory Concentration); MBC (Minimum Bactericidal Concentration); MBC/MIC: this ratio if ≥4 indicates a bactericidal activity of the analyzed compound.

Bacterial Strain	Sample	MIC mg/mL	MBC mg/mL	MBC/ MIC	Activity
Gram-negative					
*Pseudomonas* *aeruginosa* PAO1	A *	3.125	3.125	1	bactericidal
	B *	>12.5	>12.5		nd
	C *	3.125	6.25	2	bactericidal
	D *	9.9 × 10^−5^	9.9 × 10^−5^	1	bactericidal
	E *	1.7 × 10^−5^	1.7 × 10^−5^	1	bactericidal
*Escherichia* *coli* ATCC 25922	A *	1.56	1.56	1	bactericidal
	B *	>12.5	>12.5		nd
	C *	3.12	6.25	2	bactericidal
	D *	2.3 × 10^−5^	4.6 × 10^−5^	2	bactericidal
	E *	7 × 10^−6^	7 × 10^−6^	1	bactericidal
Gram-positive					
*Staphylococcus* *aureus* ATCC 6538P	A *	3.125	3.125	1	bactericidal
	B *	6.25	>12.5		nd
	C *	3.125	6.25	2	bactericidal
	D *	1.9 × 10^−4^	1.9 × 10^−4^	1	bactericidal
	E *	3.2 × 10^−5^	3.2 × 10^−5^	1	bactericidal
*Listeria* *monocytogenes* ATCC 7644	A *	0.78	0.78	1	bactericidal
	B *	12.5	>12.5		nd
	C *	3.125	6.25	2	bactericidal
	D *	9.9 × 10^−5^	9.9 × 10^−5^	1	9.9 × 10^−5^
	E *	7 × 10^−5^	7 × 10^−5^	1	1.7 × 10^−5^

* (A) Withaferin A; (B) aqueous extract and (C) hydroalcoholic extract of *W. somnifera* (D) Ciprofloxacin (E) Tobramycin; nd: not detected. Data were obtained from a minimum of three independent experiments.

## Data Availability

The original contributions presented in this study are included in the article. Further inquiries can be directed to the corresponding author.

## Abbreviations

ARE	*A. galanga* rhizomes extract
BL	Brown lower layer
CAS	Cycloartenol synthase
cDNA	Complementary deoxyribonucleic acid
CHCl_3_	Chloroform
CRC	Colorectal cancer
CTRL	Control condition
DMSO	Dimethyl sulfoxide
DMEM	Dulbecco’s Modified Eagle Medium
DWARF1	Sterol C-24 reductase
FC	Fold change
FBS	Fetal bovine serum
HPLC	High-performance liquid chromatography
IC_50_	Half-maximal inhibitory concentration
LB	Luria–Bertani medium
M	Methanolic extract
MBC	Minimum bactericidal concentration
MIC	Minimum inhibitory concentration
MTT	3-(4,5-Dimethylthiazol-2-yl)-2,5-diphenyltetrazolium bromide assay
NMR	Nuclear magnetic resonance
PAO1	*Pseudomonas aeruginosa* strain PAO1
PCSLE	Pressurized Cyclic Solid–Liquid Extraction
qRT-PCR	Quantitative reverse-transcription polymerase chain reaction
RNAi	RNA interference
RPM	Revolutions per minute
RT-PCR	Reverse transcription polymerase chain reaction
SEM	Standard error of the mean
SH-SY5Y	Human neuroblastoma cell line
SMT1	Sterol-C24-methyltransferase type 1
TLC	Thin-layer chromatography
UV	Ultraviolet detection
